# 1,135 Genomes Reveal the Global Pattern of Polymorphism in *Arabidopsis thaliana*

**DOI:** 10.1016/j.cell.2016.05.063

**Published:** 2016-07-14

**Authors:** The 1001 Genomes Consortium, Carlos Alonso-Blanco, Jorge Andrade, Claude Becker, Felix Bemm, Joy Bergelson, Karsten M. Borgwardt, Jun Cao, Eunyoung Chae, Todd M. Dezwaan, Wei Ding, Joseph R. Ecker, Moises Exposito-Alonso, Ashley Farlow, Joffrey Fitz, Xiangchao Gan, Dominik G. Grimm, Angela M. Hancock, Stefan R. Henz, Svante Holm, Matthew Horton, Mike Jarsulic, Randall A. Kerstetter, Arthur Korte, Pamela Korte, Christa Lanz, Cheng-Ruei Lee, Dazhe Meng, Todd P. Michael, Richard Mott, Ni Wayan Muliyati, Thomas Nägele, Matthias Nagler, Viktoria Nizhynska, Magnus Nordborg, Polina Yu. Novikova, F. Xavier Picó, Alexander Platzer, Fernando A. Rabanal, Alex Rodriguez, Beth A. Rowan, Patrice A. Salomé, Karl J. Schmid, Robert J. Schmitz, Ümit Seren, Felice Gianluca Sperone, Mitchell Sudkamp, Hannes Svardal, Matt M. Tanzer, Donald Todd, Samuel L. Volchenboum, Congmao Wang, George Wang, Xi Wang, Wolfram Weckwerth, Detlef Weigel, Xuefeng Zhou

**Affiliations:** 1 Max Planck Institute for Developmental Biology, Spemannstrasse 35, 72076 Tübingen, Germany

**Keywords:** *Arabidopsis thaliana*, 1001 Genomes, glacial refugia, GWAS, population expansion

## Abstract

*Arabidopsis thaliana* serves as a model organism for the study of fundamental physiological, cellular, and molecular processes. It has also greatly advanced our understanding of intraspecific genome variation. We present a detailed map of variation in 1,135 high-quality re-sequenced natural inbred lines representing the native Eurasian and North African range and recently colonized North America. We identify relict populations that continue to inhabit ancestral habitats, primarily in the Iberian Peninsula. They have mixed with a lineage that has spread to northern latitudes from an unknown glacial refugium and is now found in a much broader spectrum of habitats. Insights into the history of the species and the fine-scale distribution of genetic diversity provide the basis for full exploitation of *A. thaliana* natural variation through integration of genomes and epigenomes with molecular and non-molecular phenotypes.

## Highlights

• The genomes of 1,135 naturally inbred lines of *Arabidopsis thaliana* are presented
• Relict populations that continue to inhabit ancestral habitats were discovered
• The last glacial maximum was important in structuring the distribution of relicts
• This collection will connect genotypes and phenotypes on a species-wide level

## Graphical Abstract

**Figure:**
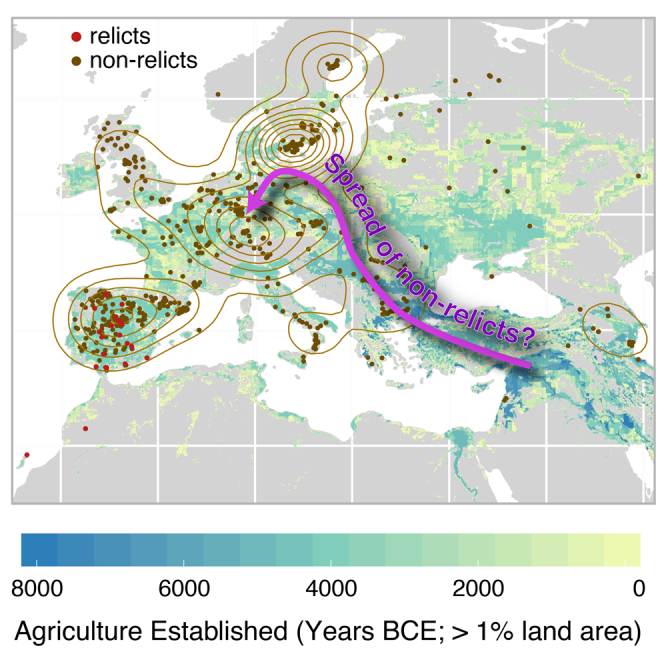

## Teaser

Genomic sequencing analysis of over 1,000 natural inbred lines of *Arabidopsis thaliana* reveals its global population structure, migration patterns, and evolutionary history and provides a rich genetic resource for studying phenotypic variation and adaptation.

## Introduction

*Arabidopsis thaliana* remains at the forefront of modern genetics. Decades of work have not only established much of what we know about the physiology and development of plants but also provided insight into how wild populations adapt to biotic and abiotic environments. Few systems share the key advantage of *A. thaliana* for GWAS or complementary forward genetics approaches: the ready availability of a large collection of naturally inbred lines (accessions) that are products of natural selection under diverse ecological conditions. This makes it possible to link genotypes and phenotypes to fitness effects in the laboratory and the field (Aranzana et al., 2005, Atwell et al., 2010, Fournier-Level et al., 2011, Hancock et al., 2011). By adding molecular data for genetically identical individuals—e.g., RNA expression or epigenetic marks— underlying mechanisms can be elucidated much more easily than in other multicellular species.

The fundamental resource for this research program is a set of accessions with complete genome sequences, collected from different locales. Systematic characterization of genome-wide polymorphism in *A. thaliana*, paralleling efforts in humans (Birney and Soranzo, 2015), began with a description of linkage disequilibrium (Nordborg et al., 2002) and population structure in 96 accessions (Nordborg et al., 2005). This was followed by a whole-genome map of deletions and SNPs in 20 global accessions (Clark et al., 2007), which in turn was the basis of a 250k SNP array with multiple markers in each haplotype block (Kim et al., 2007). This array was used to genotype the RegMap collection of 1,307 diverse accessions (Horton et al., 2012). Concurrent with the application of short read sequencing to human genomes, the first *A. thaliana* genomes were resequenced (Ossowski et al., 2008), soon followed by the analysis of larger collections (Cao et al., 2011, Gan et al., 2011, Long et al., 2013, Schmitz et al., 2013). Similar efforts have led to large-scale surveys of sequence diversity in other plants, mostly crops (Chia et al., 2012, Huang et al., 2012, Lin et al., 2014, 100 Tomato Genome Sequencing Consortium, 2014, 3000 Rice Genomes Project, 2014, Zhou et al., 2015).

We extend these efforts with 1,135 *A. thaliana* accessions from a worldwide hierarchical collection. There were several motivations for the current study: to quantify genome variation in a larger and more representative sample of accessions; to investigate the demographic history of the species; to identify features that make specific geographic or genetic subsets particularly well suited for forward genetics, field experiments and selection scans; and to provide a powerful GWAS platform. Previous studies had shown that the ability to detect footprints of selection depended greatly on the sample (e.g., Cao et al., 2011, Long et al., 2013, Huber et al., 2014). Similarly, while GWAS have identified common alleles with major effects from as few as 96 accessions (Aranzana et al., 2005, Atwell et al., 2010), a much larger sample is required for most traits. The SNP-genotyped RegMap panel (Horton et al., 2012) provided such a collection but did not efficiently capture all SNPs and structural variants. Fully sequencing this collection would be of limited benefit, as one could accurately impute the missing data by sequencing a subset. We therefore assembled a set of accessions that sufficiently overlap the RegMap panel for imputation of variants in all lines. The combined collection constitutes a first-rate resource for determining how genetic variation translates into phenotypic variation.

## Results and Discussion

### The Sample

**Figure 1:**
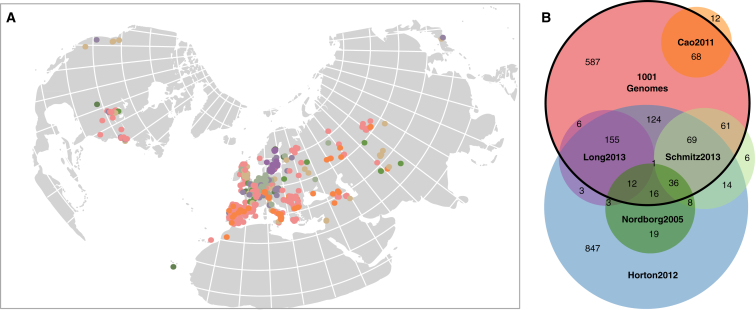
Origins of the 1001 Genomes Accessions (A) Collection locations of the 1001 Genomes accessions by diversity set (colors correspond to Venn diagram in B). (B) Relationships between 1001 Genomes accessions and other *A. thaliana* diversity sets (Nordborg et al., 2005, Cao et al., 2011, Horton et al., 2012, Long et al., 2013, Schmitz et al., 2013).

We selected accessions for Illumina short read sequencing with several objectives in mind. We sought to cover the global distribution of *A. thaliana* more evenly than the RegMap panel (Horton et al., 2012) while including large regional collections of particular interest from ecological and evolutionary perspectives, notably from Sweden and the Iberian Peninsula (Figure 1A). We also wanted to better sample interesting regions based on prior knowledge of the population structure of the species, such as North America and Central Asia (Sharbel et al., 2000, Nordborg et al., 2005, Schmid et al., 2003, Beck et al., 2008, Platt et al., 2010, Cao et al., 2011, Brennan et al., 2014, Long et al., 2013). Our collection is hierarchical, with a range of geographic distances between nearest neighbors, and a few very densely sampled locales. Most accessions had been genotyped with 149 genome-wide intermediate-frequency SNP markers (Platt et al., 2010) to avoid sequencing identical individuals.

After filtering (described below), we retained sequences of 413 RegMap and 722 new lines, for a total of 1,135 accessions with whole-genome information (see the Data Release section). These 1,135 lines are the focus of this paper; the imputed RegMap set will be described in another paper. Together, the RegMap and 1001 Genomes samples include 2,029 natural *A. thaliana* accessions with high-quality polymorphism data (Figure 1B).

The genomes presented here integrate previously published subsets (Cao et al., 2011, Gan et al., 2011, Horton et al., 2012, Long et al., 2013, Schmitz et al., 2013, Hagmann et al., 2015; Figure 1B). All accessions are available from the stock centers, and we have generated an accession list (see Data Release section) that unifies previous naming schemes and provides provenance information. Our intention is for this collection to remain actively curated as ever more accurate genomes are produced and a wide range of phenotypic data are generated (not only by us, but also by the community— see www.1001genomes.org for information on how to contribute).

### The Genomes

A range of Illumina platforms were used across several sequencing centers and over several years, so we instituted stringent quality controls to pare an initial set of over 1,200 sequenced genomes to a final set of 1,135 (see Data Release section). The data are the intersection of the MPI (SHORE) and GMI (GATK) pipelines, independently validated in our pilot studies (Cao et al., 2011, Long et al., 2013). An average of 100 Mb (84%) per line were called against the TAIR10 reference genome (119 Mb). The missing positions differ greatly between lines, such that only 2% of the reference genome lack calls entirely. Based on comparisons with one long read (Pacific Biosciences) and three short read (Illumina) based de novo genome assemblies, we estimate that fewer than 3% of SNP calls are erroneous (i.e., should be reference instead) independently of dataset coverage, with the vast majority being singletons. Over 98% of genotype calls were correct at SNP sites, and only 1.5% of SNPs were mistakenly called as reference (Supplemental Experimental Procedures). We emphasize, however, that this calculation ignores SNPs missed because they are in the vicinity of structural variants, which are difficult to assess with short read technology.

After filtering, the nuclear genomes contained 10,707,430 biallelic SNPs and 1,424,879 small-scale indels (up to 40 bp). This represents one variant on average every 10 bp of the single copy genome, which is the densest variant map for any organism, including the most recent release of the 1000 Genomes Project for humans (1000 Genomes Project Consortium, 2015). 2,842 biallelic SNPs were called in chloroplast genomes and 824 in mitochondrial genomes. The complete data are available as VCF files and as FASTA pseudogenomes (see Data Release section). We also developed web applications to subset the full genome VCF or pseudogenome files and extract data on a selection of genomes and/or specific loci as well as a “Strain ID” application, with which users can identify the genomes in our sample that are most closely related to a newly sequenced genome (see Data Release section). As with all short read data, we advise caution in using our pseudogenomes with applications in which the contiguity of DNA sequence is critical, for example, in the generation of PCR primers. Finally, we are committed to supporting the community in developing additional applications that make use of these data.

### Genome-wide Association Studies

**Figure 2:**
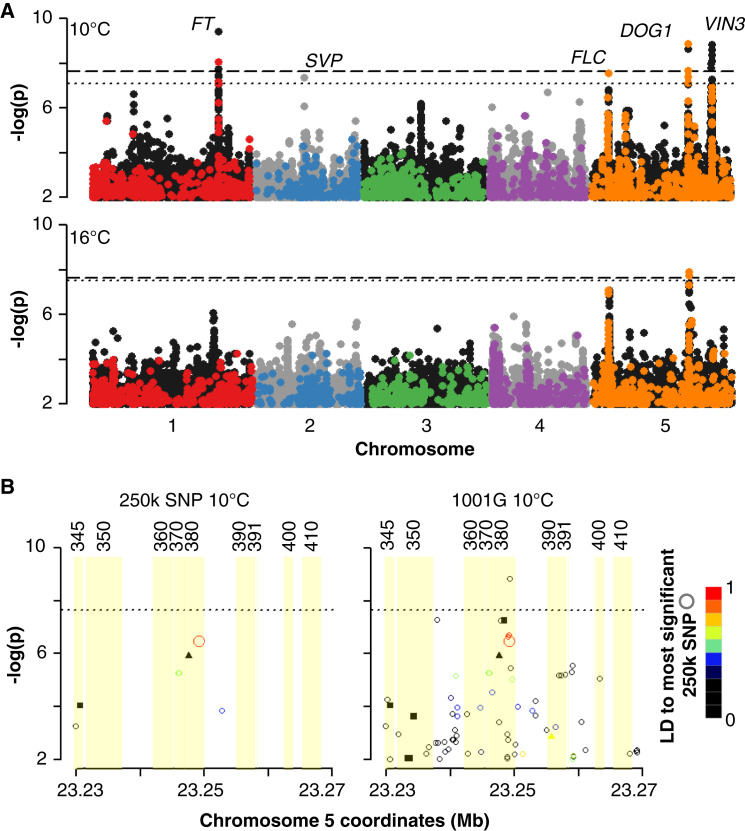
Comparison of GWAS for Flowering Time Using Full Genome Variants and RegMap SNPs (A) Long day flowering time GWAS with four replicates in 1,003 (10°C) and 971 (16°C) lines. Horizontal lines represent 5% significance thresholds corrected for multiple testing using Bonferroni (dashed) and permutations (dotted). Black and gray dots are all 1001G variants, colored dots the subset also found on the RegMap 250k array. (B) Comparison of GWAS results near flowering time regulator *VIN3* (At5g57380) with the 180k biallelic SNPs (MAF > 0.03) from the 1001 Genomes full-genome set present on the RegMap 250k array. Numbers above are regional gene identifiers, e.g., “345” = “At5g57345.” Shapes denote SNP annotation: circles are non-coding; squares are synonymous; triangles are non-synonymous. Colors represent linkage disequilibrium to the top-ranked SNP in the 250k data.

A major motivation for sequencing a large collection of accessions is to enable GWAS with nearly complete genotype information. For comparison with the RegMap data, we measured flowering time under different environments (10°C and 16°C) in our collection and performed GWAS. We note first that there is little reason not to use full genome data, as permutation-based multiple-comparison thresholds (He et al., 2014, Abney, 2015) can be used to minimize the statistical cost of additional markers (Figure 2A). The chromosome 5 peak at ∼23.25 Mb nicely illustrates the advantage of the full genome data (Figure 2B). Although clearly visible using the 192,498 biallelic variants from the 250k SNP array (Horton et al., 2012), not a single SNP reaches genome-wide significance, and the peak might well have been ignored, were it not for the fact that the most significant SNP lies in an intron of the flowering time regulator *VIN3* (Sung and Amasino 2004). In contrast, the full data clearly reveal a significant peak. Notably, the lead SNP in this peak is not in linkage disequilibrium with the tag SNP from the 250k SNP array, even though the two variants are only 60 bp apart.

The remaining peaks (Figure 2A) contain the flowering regulators *FT*, *SVP*, *FLC*, all previously linked to flowering time variation (Schwartz et al., 2009, Méndez-Vigo et al., 2013, Li et al., 2014), and the dormancy regulator *DOG1*, recently shown to affect also flowering time (Huo et al., 2016). As previously noted (Atwell et al., 2010), linkage disequilibrium is normally too extensive to directly pinpoint the causative genes or variants with GWAS alone. For example, the peak on chromosome 2 that contains *SVP* also includes At2g22590, which codes for an UDP-glucosyltransferase, a family of proteins linked to the control of *FLC* expression (Wang et al., 2012).

### Population Structure

**Figure 3:**
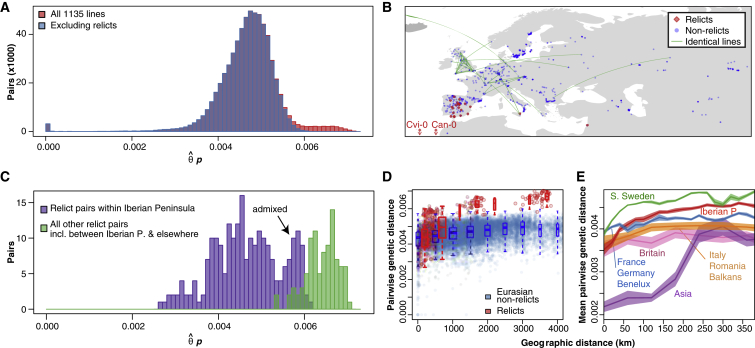
Genetic and Geographic Distances between Accessions (A) The trimodal distribution of pairwise genetic distances among accessions. The mode near zero reflects very close relationships of nearly identical accessions. The mode near 0.007 includes comparisons between relicts and non-relicts. (B) Geographic locations of relicts (red) and non-relicts (blue) in Eurasia and North Africa, with pairs of nearly identical accessions at least 1 km apart connected by green lines. (C) Genetic distances of relict pairs. Pairwise distances between Iberian relicts are of similar magnitude as distances between global non-relicts (see Figure 3A), while the distances between relict groups from different geographic regions are higher. The second mode of high divergence for Iberian relicts is due to accessions admixed with non-relicts. (D) Genetic distance increases globally with geographic distance for relicts but for non-relicts only over short distances. Horizontal lines indicate median, boxes include second and third quartiles, and whiskers indicate 1.5 times interquartile range. (E) At regional scales, the rate at which genetic distance scales with geographic distance varies greatly among geographic regions for non-relicts. For each geographic region, the plot shows the genetic distance in bins of increasing geographic distance (a bin-distance of 20 km was used for S. Sweden, Iberian Peninsula, France/Germany/Benelux and 60 km bins were used for Asia, Italy/Romania/Balkans, and Britain because of uneven sampling). The shaded areas show 95% confidence intervals calculated using the ciMean function from the R package lsr. See also Figures S1, S2, and S3.

GWAS provide insights into the genetic basis of natural variation. To interpret such variation, it is essential that we understand the evolutionary history of a species. For an organism such as *A. thaliana*, the simplest population genetics model is strict isolation by distance (IBD), under which the genetic distance between individuals reflects only geographic distance. This model does not fit, as the peaks of pairwise differences do not reflect geography (Figure 3A). One extreme encompasses groups of (nearly) identical individuals corresponding to inbred lineages, a result of selfing. This includes 78 North American accessions, with several smaller clusters of three to seven members, and 40 pairs of accessions that differ by fewer than 1k SNPs (Figure 3B). 60 additional pairs differ by fewer than 50k SNPs, much less than the median of 439,145 for all comparisons. Excluding North American and British accessions, ∼80% of these nearly identical pairs were collected within 1 km of each other, most within a few meters. It remains unclear whether the remaining pairs represent true long distance migration, rather than mis-assignment or mix-ups after collection (see Supplemental Experimental Procedures).

Both North America and the British Isles show evidence of recent long-range dispersal (Platt et al., 2010, Horton et al., 2012). While North America harbors a single lineage due to very recent colonization (Hagmann et al., 2015), the British Isles contains numerous widely spread genotypes, suggestive of a more ancient and gradual colonization. The median geographic distance between nearly identical British pairs is 303 km, and only 1 of 40 nearly identical pairs was collected from the same site. While some pairs may reflect labeling errors after collection, close genetic relationships are also observed among more diverged but still rather similar pairs of British accessions, supporting that they are the product of recent gene flow.

At the other end, extreme pair-wise divergences (Figure 3A) are seen with 26 accessions, including 22 from the Iberian Peninsula, and one line each from the Cape Verde Islands, Canary Islands, Sicily, and Lebanon (see Data Release section). We refer to these accessions as “relicts.” The 22 Iberian relicts are no more different from each other than are pairs of non-relicts (Figure 3C). The remaining four relicts stand apart from each other and from all other accessions.

By genetic distance, our 1,135 accessions thus comprise six diverged groups: four relict groups with a single line each; one relict group of 22 Iberian accessions; and the majority group of 1,109 accessions. It should be noted that accessions Mr-0 from Italy and Tnz-1 from Tanzania also were extremely diverged, but their sequences failed quality controls and were not included in the final 1,135 accessions. Re-sequencing confirmed that Mr-0 (closely related to Sicilian relict Etna-2) and two further Tanzanian accessions, Tanz-1 and Tanz-2, are relicts. Their sequences will be available in the next data release.

**Figure S1:**
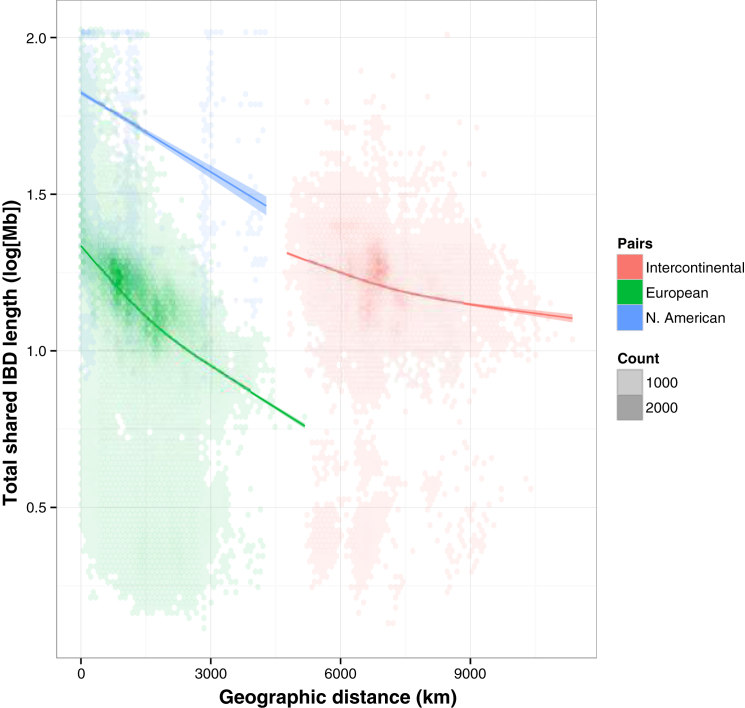
Overall Relationship between Total Length of Identity-by-Descent Segments and Geographical Distance in Kilometers for Each Pair in Three Groups, Related to Figure 3 In the US, many pairs share IBD segments that total over 85 Mb; in Europe, this is the case for only 0.05% of pairs, with the vast majority of pairs having IBD sharing in the range of 15-25 Mb. The minimal IBD length threshold was 10 kb. Intermediate values of intercontinental comparisons are consistent with a recent colonization of North America from European ancestors. Hexagonal bins of density, with General Additive Model predictions (k = 3, solid lines) and the 95% CI of these predictions (shaded regions).

The geographic distribution of relicts and non-relicts (Figure 3B) confirms that a naive IBD model cannot hold. For example, Iberian non-relicts are more closely related to accessions from Kazakhstan than to Iberian relicts. Moreover, while relicts show strong IBD on all geographic scales, non-relicts have a similarly clear pattern only over short distances (Figure 3D), as expected if they had spread rapidly to occupy their current, global range, while the relicts had largely stayed put. On a regional scale, there is considerable geographic variation in the strength of IBD among the non-relicts, indicating that the history of colonization is complex (Figure 3E). The existence of outlier accessions, such as Cvi-0 and Mr-0, has been noted before (Nordborg et al., 2005); it is now clear that there are many such accessions, and that they can be common locally. Our data also confirm that the colonization of North America was recent and rapid (Platt et al., 2010, Horton et al., 2012, Hagmann et al., 2015). In addition to groups of nearly identical individuals, 47% of North American pairs exhibit extensive haplotype sharing (total identity-by-descent length over 85 Mb, as inferred using Beagle and GERMLINE, Figure S1) (Browning and Browning, 2009, Gusev et al., 2009), indicating recent mixing among a limited number of initial immigrants. Conversely, European accessions have low genetic relatedness, and the extent of haplotype sharing generally decays with geographic distance.

To examine population structure in greater detail, we used ADMIXTURE (Alexander et al., 2009) to cluster the accessions. In addition to identifying most of the relicts as a genetically distinct group, this analysis breaks non-relicts into eight clusters that broadly correspond to geography (see Data Release section). We defined nine groups based on these clusters and assigned each individual to a group if more than 60% of its genome derived from the corresponding cluster. The 135 individuals not matching this criterion were labeled “Admixed.” There is evidence for admixture between the relict and non-relict groups, as two accessions initially identified as relicts, from Sicily and Lebanon, were found to be admixed. These ADMIXTURE classifications were used in all subsequent analyses.

**Figure S2:**
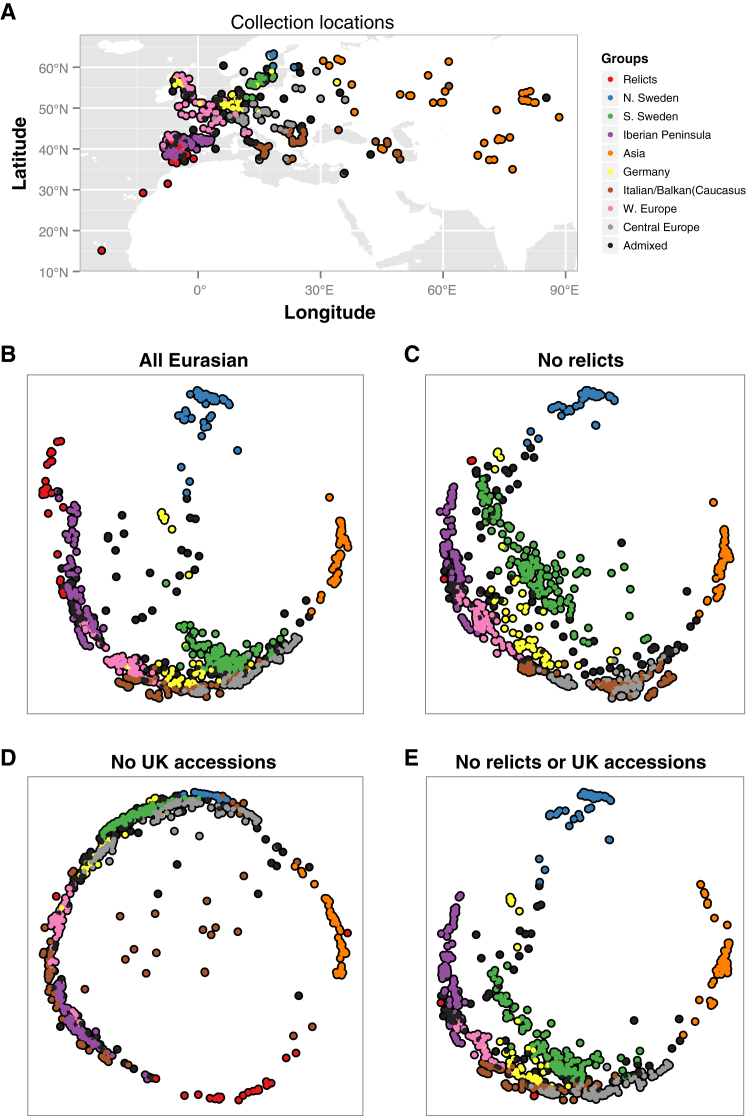
Geographic Prediction from SPA Suggests that a Simple Isolation-by-Distance Model Does Not Hold, Related to Figure 3 (A and B) Under this model, if geographic gradients of SNPs were smooth, the collection locations of the accessions should be recovered in the SPA analysis in B. They are not. Instead, we find strong spatial gradients between, and gentle gradients within groups. (C and D) The strength of these gradients is disproportionately influenced by the relict and UK accessions. (E) Finer geographic gradients of SNP variation, especially in Southern Swedish populations, are observable when relict and UK accessions are excluded. Note that unsupervised predictions from SPA analysis are translation, scale, and rotation invariant (dimensionless).

**Figure S3:**
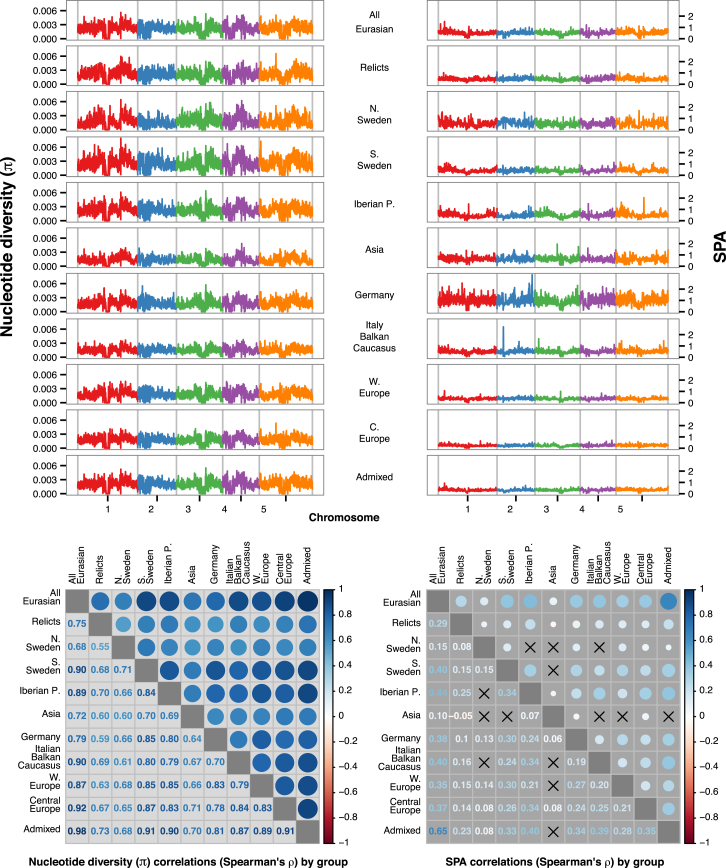
Nucleotide Diversity (π) and Spatial Gradients of Genetic Diversity (SPA) by Group in 50 kb Windows, Related to Figure 3 While overall genetic diversity across the genome is similar across genetic groups, the geographic distribution of that variability differs across groups, with the lowest associations between the North Swedish and Asian groups. Correlations that did not reach the significance threshold of p = 0.01 are marked with an “X.”

The ADMIXTURE groups do not correspond to idealized randomly mating populations. There is a regional and variable pattern of IBD (Figure 3E). Similarly, geographic locality prediction using SPA (Yang et al., 2012) demonstrates the existence of population structure both within and between groups (Figure S2) and highlights the variability in IBD (Figure S3).

**Figure 4:**
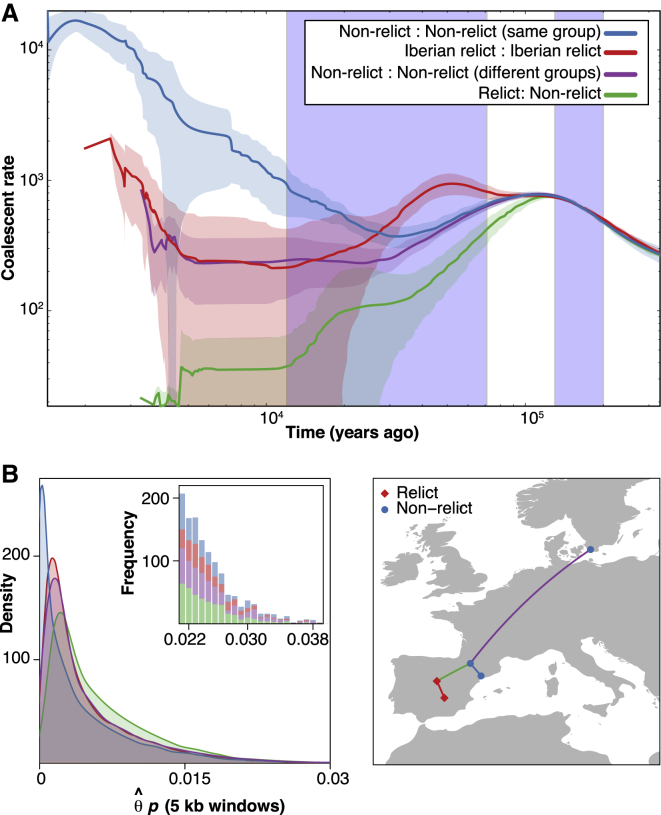
Evidence for the Importance of the Last Glacial Maximum in Structuring Historic and Modern Distribution of Relict and Non-relict Groups (A) Coalescence rates over time for pairs of individuals from different ADMIXTURE groups, inferred using MSMC. Comparisons are between non-relicts from the same group (blue), Iberian relicts (red), non-relicts from different groups (purple), and relicts and non-relicts (green). The latter also includes comparisons of relicts from different geographic regions, which look similar to relict—non-relict comparisons. Solid lines indicate means, shading standard deviations. Between 49 and 62 random pairs were used. Light blue vertical bars show the last four glacial periods. (B) Left, distributions of pairwise nucleotide diversity in 5-kb windows for four selected pairs of accessions. Colors indicate provenance of accessions, shown on right. Inset, counts in the extreme tail of the distributions. See also Table S1.

To elucidate the historical processes that have shaped extant diversity, we estimated the distribution of coalescence times for the different populations using MSMC (Schiffels and Durbin, 2014). The results suggest that glacial refugia are largely responsible for present population structure (Figure 4A). Coalescence rates are an indication of relatedness, with higher rates indicating closer average relatedness (and smaller effective population size). Since the last glaciation, coalescence rates within non-relict ADMIXTURE groups were much higher than for Iberian relicts, or between members of different non-relict groups, and coalescence rates between relicts and non-relicts were essentially zero. The rate of coalescence between relicts and non-relicts was also lower than the other rates during the last glaciation, indicating that they were isolated from each other during this period. At the same time, the rate of coalescence among Iberian relicts was high, indicating a local bottleneck, with only slight differences in coalescence rate within and between non-relict groups, consistent with these groups being the product of post-glacial expansion.

In contrast, current population structure is not reflected in the rate of coalescence before the last glaciation; there has since been sufficient migration and time to erase all traces of earlier population structure. The distribution of highly diverged haplotypes at individual loci in the genome is thus independent of present population structure. The first polymorphism study in *A. thaliana*, with *ADH*, noted already the presence of surprisingly diverged haplotypes, and interpreted it as evidence for balancing selection (Hanfstingl et al., 1994). Many realized that the phenomenon was common and that deep population structure must be responsible (Aguadé, 2001, Nordborg et al., 2005, Wright and Gaut, 2005). However, the population structure required to account for pairwise sequence divergence of several percent at individual loci, compared to a genome-wide average of 0.5%, must be far older than the most recent glaciation. Thus, while recent coalescence times, as reflected in low pairwise sequence divergence, are more common in within-group comparisons (Figure 4B), the tails of extreme values look very similar for within- and between-group comparisons (Figure 4B, inset).

It is important not to exaggerate the divergence between relicts and non-relicts. In a survey of four-sample gene genealogies between the Col-0, Ler-0, and two random Iberian relicts, 26% place Col-0 closer to one relict than to Ler-0, rather than the two non-relicts together (Table S1). Two additional four-sample analyses, one with the outgroup *A. lyrata*, Col-0, and two relicts, and the other with *A. lyrata*, one relict, Col-0 and another non-relict, produced similar patterns. While genes with the expected topology were significantly more common (43% and 53%, p < 0.001), many genes supported the alternative topology of a non-relict being closest to a relict.

### Footprints of Selection in the Genome

The last glacial maximum was followed by pronounced expansion of the global *A. thaliana* population. It is therefore natural to search for footprints of selection related to adaptation to new environments, especially to climate, which varies considerably across the species range (Hancock et al., 2011). We examined correlations of genetic variants with six climate variables that capture variability in current temperature and precipitation using a mixed model that controls for genome-wide relatedness across samples (Zhou and Stephens, 2012). Twenty SNPs are significantly correlated with precipitation-related variables, at a False Discovery Rate of 5% (Table S2). Three associations are characterized by a much higher derived allele frequency in the Iberian relicts than in the general population, possibly indicative of local adaptation from new mutations. One affects the *ERF1* drought response regulator (Cheng et al., 2013). *ERF1* is also involved in resistance to several fungal pathogens (Berrocal-Lobo and Molina, 2004), as is *MLO11* (Acevedo-Garcia et al., 2014), which is located near two of the other variants. The connection to drought response and fungal defense suggests that selection could be due to tradeoffs between abiotic and biotic stress.

**Figure 5:**
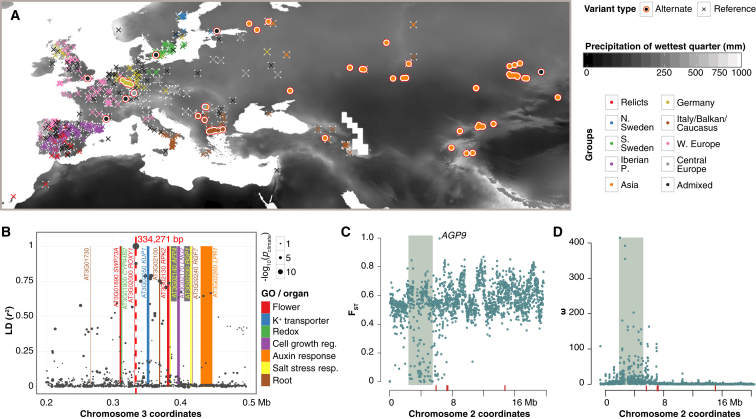
Footprints of Selection (A) Distribution of accessions containing the reference or alternate variant for a locus strongly associated with precipitation in the wettest quarter. The alternate allele is most frequent in the Asian group, but it is also present in other groups. (B) A climate associated and spatially disjunct SNP (red dashed line), located in a region densely populated with genes affecting traits such as root growth, salt tolerance, flowering, and detoxification. (C) The distribution of maximum *F*_ST_ scores in 10-kb windows along chromosome 2. The centromere is shaded, and the locations of NLR-containing disease resistance genes are in red. (D) The distribution of ω statistics in 10-kb windows along chromosome 2. Labels as in Figure 5C. See also Figures S4 and S5 and Tables S2, S3, and S4.

Strong local adaptation may create abrupt geographical changes in allele frequency. We used SPA (Yang et al., 2012) to search for SNPs showing this pattern, and intersected these SNPs with climate GWAS hits to identify variants with local adaptation to climate. Spatial and climatic distributions of genetic variants are intertwined with population structure (Figure S3), so almost no significant variants remain once population structure is taken into account. A single variant associated with precipitation in the wettest quarter also shows a significant geographic gradient (Figure 5A). This variant is in a genomic region densely populated with genes that have been implicated in root development and metabolism, flowering time and flower development, salt tolerance, and detoxification (Figure 5B).

**Figure S4:**
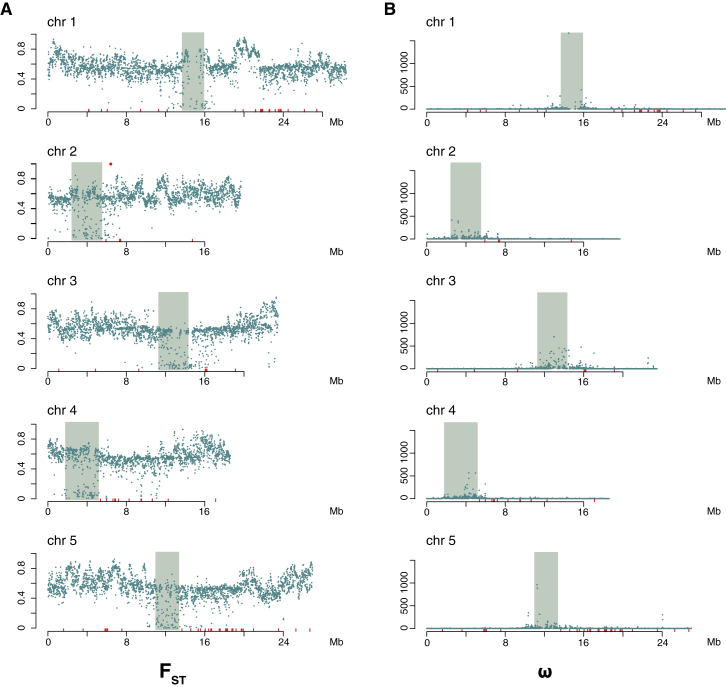
Genome-wide *F*_ST_ and ω for Each Chromosome, Related to Figure 5 (A) Distribution of maximum *F*_ST_ scores in 10-kb windows. The centromere is shaded in each figure, and the locations of NB-LRR genes (“resistance” or *R* genes) are shown in red. (B) Distribution of ω-statistic in 10-kb windows. Labels as in A. On each chromosome, the lowest genome-wide *F*_ST_ values, and largest estimates of the ω-statistic, are near the centromeres, which suggests that selective sweeps or background selection are common in these regions of the genome.

Because the strategies above attempt to eliminate false positives from population structure, it is difficult to detect variants under population-specific selection. To identify such genes, we calculated *F*_ST_ between admixture groups for all SNPs (Weir and Cockerham, 1984). The most diverged region is on chromosome 2 at 6.401 Mb, overlapping the gene encoding *AGP9*, which has not been linked to adaptive processes before (Figures 5C and S4A). Regions adjacent to centromeres exhibit the lowest *F*_ST_ values. In agreement with previous results (Long et al., 2013), these regions contain excessive linkage disequilibrium (ω, Figures 5D and S4B), which suggests that they have been shaped by selective sweeps or background selection.

In addition to these global patterns, we identified loci that may contribute to adaptive differences between Iberian relicts and non-relicts. We paired each relict with the geographically closest non-relict (Table S3). Over 100 variants have diverged between the two groups, including several in or near *EIN2*, a development and stress regulator (Alonso et al., 1999), and *AP2*, which is important for flower and seed development (Licausi et al., 2013). Additional genes with differentially fixed variants are *LUG* and *SLK1*, which encode transcriptional co-repressors that interact biochemically and genetically with each other and with *AP2* (Sridhar et al., 2004, Bao et al., 2010). Finally, a deeply diverged region around 18.796 Mb on chromosome 2 includes two flowering time regulators, *AGL6* and *SOC1* (Samach et al., 2000, Huang et al., 2013). As expected from these candidates of selection, the top biological processes (GO terms) strongly overrepresented in these results are “flower development” and “ABA-related activities” (Table S4). Consistent with differentiation in flowering time, relicts flower in 10°C long days on average 21 days later than their nearest non-relicts (t = 4.69, df = 41; p = 3 × 10^−5^), suggesting that life-history differences contributed to the spread of non-relicts.

**Figure S5:**
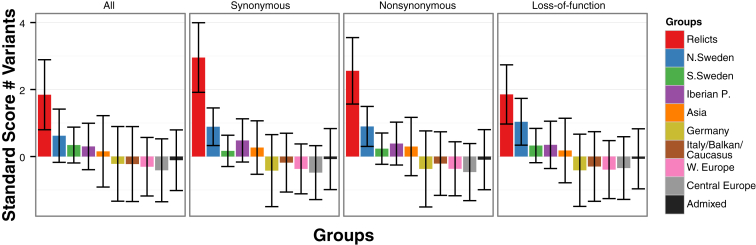
Variants by Type and Group, Related to Figure 5 Mean and standard deviation of the standard score (Z-score) of the number of variants of each type, by group. Relicts show the greatest normalized number of variants, especially of synonymous variants. Bars indicate means, whiskers represent one standard deviation.

Demographic history can affect the efficacy of selection, and mutations that are likely to be deleterious are common in *A. thaliana*, especially in marginal populations (Cao et al., 2011). We therefore predicted the impacts of coding sequence variants in different genetic groups using SNPeff (Cingolani et al., 2012). Most genes, 27,525, contained at least one variant likely to change protein function, with 17,692 having at least one high-impact variant. On average, 440 genes per accession, for a total of 15,060 genes, had at least one variant predicted to inactivate the gene, although this is likely an overestimate, as it does not account for compensatory mutations or different transcript isoforms (Gan et al., 2011, Schneeberger et al., 2011, Long et al., 2013). Relicts have proportionally the most genes with potentially deleterious mutations, consistent with a reduced efficiency of selection in the relicts due to small effective population size, with the caveat that mapping to a non-relict reference may again lead us to overestimate such variants (Figure S5).

### Conclusions

#### *The Natural History of* A. thaliana

The exquisite detail with which we have characterized the spatial pattern of polymorphism in *A. thaliana* has clarified prior hypotheses and revealed surprising aspects of the species’ history. In particular, the crucial importance of the last ice age has come into much sharper relief (Sharbel et al., 2000, Nordborg et al., 2005, Schmid et al., 2003, Beck et al., 2008, François et al., 2008, Picó et al., 2008). The picture that emerges is that modern *A. thaliana* is a complex mixture of survivors from multiple glacial refugia, with population expansion having strongly favored the descendants of a specific refugium, possibly as a result of human activity. Under this model, the “relict” accessions are simply those that survived this expansion/invasion. Several lines of evidence support this interpretation.

**Figure 6:**
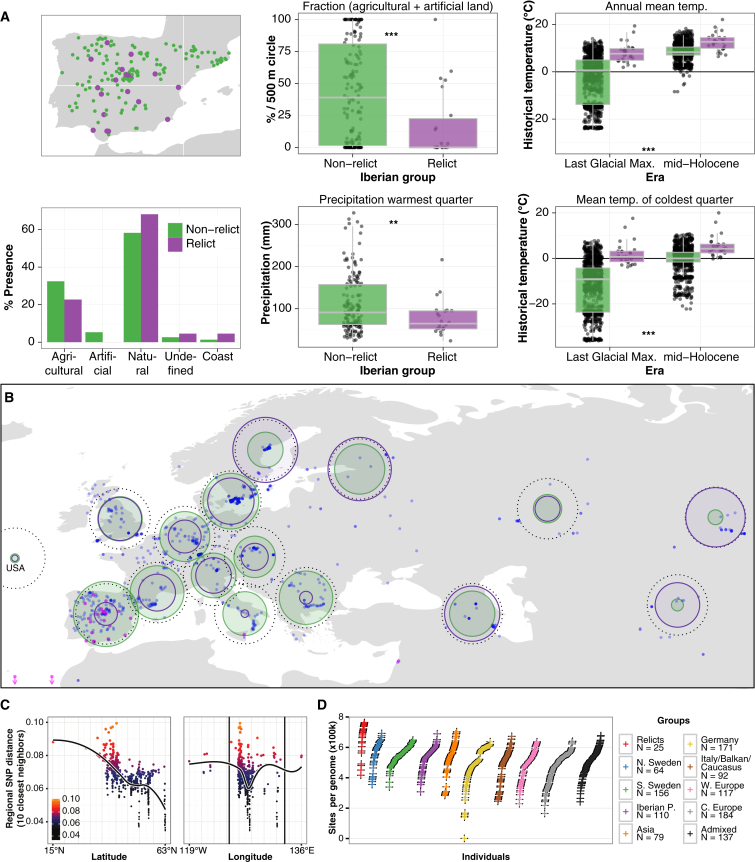
Local Genetic Diversity in Different Regions and Groups (A) Current land use, current and paleoclimate for relicts and non-relicts. Relicts are purple (^∗∗^p < 0.01; ^∗∗∗^p < 0.001). Horizontal lines indicate median, boxes include second and third quartiles, and whiskers indicate 1.5 times the inter-quartile-range. (B) The geographic distribution of average pairwise distance (π) and Tajima’s D. Sizes of the green circles indicate regional π (range from 0.002 [USA] to 0.006 [Iberian Peninsula]). Dotted circles indicate the global value, 0.006. Size of purple circles represent the regional values of Tajima’s D (range from −1.01 [Northern Sweden] to −2.08 [USA], global value −2.04). Blue dots indicate sampling sites. (C) Regional diversity as a function of latitude or longitude. (D) Rank ordered distribution of non-private variants in each accession by ADMIXTURE group, offset to show density. See also Figure S6.

**Figure S6:**
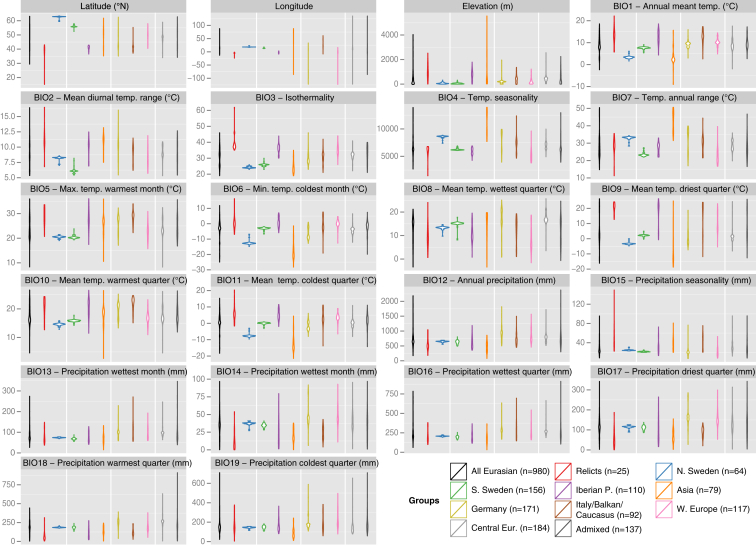
Climatic and Geographic Representation of Samples within Groups, Related to Figure 6 While the distribution of climate within each group is generally in proportion to the distribution of geography (latitude, longitude, and elevation), some are not. For example, although the Asian accessions are widely distributed, the range of precipitation experienced by these accessions is surprisingly narrow. Note that a few non-Eurasian accessions were nevertheless assigned to admixture groups, and are included in these distributions. Violin plots show probability densities within admixture groups for each geoclimatic variable.

The pattern of isolation-by-distance suggests that relict populations have been relatively stationary while the non-relicts’ range rapidly expanded (Figure 3D). Consistent with this model, the climate at the relict locations has changed much less since the last glacial maximum than where modern non-relicts are found (Figures 6A and S6). The Iberian Peninsula is especially interesting, given the presence of a large number of relicts interspersed with non-relicts (Figure 3C). Although relicts are widely distributed there, they are restricted to a very specific environment characterized by old oak and pine forests, high climate seasonality, high temperatures, and low rainfall (Figure 6A). Iberian relicts correspond to a genetic lineage that has been previously identified in the southwestern Mediterranean region, supporting the idea that they survived in a glacial refugium in North Africa (Brennan et al., 2014). In contrast, non-relicts are found more often in agricultural and urban areas, consistent with expansion of non-relicts having been associated with human activity, and with the relative rarity of relicts reflecting destruction of undisturbed habitats.

The source of the non-relicts, which comprise most modern *A. thaliana* individuals, remains obscure. The Iberian Peninsula has the largest regional diversity, and Mediterranean regions tend to be more diverse than other regions (Figure 6B). There is a gradient of decreasing diversity from south to north (Figure 6C), as expected after a range expansion from southern glacial refugia (Petit et al., 2003). However, this pattern is likely due to admixture between relicts and invading non-relicts in these regions (high diversity in the Iberian Peninsula almost certainly is) and does not reveal the origin of the invaders. Indeed, omitting relict and admixed accessions, it would be easy to come to the conclusion that the center of diversity is southern Sweden and that diversity decreases from north to south across the entire range. The relatively high diversity seen in southern Sweden (Figure 6D), and also in Russia (Figure 6B) may similarly be the result of admixture, in this case between the original post-glacial colonizers and more recent weedy varieties that accompanied the spread of agriculture, perhaps giving rise to the higher values of Tajima’s D in these regions. Resolving this issue using only contemporary collections will be difficult.

One pattern that does seem clear is that longitudinal gradients of regional diversity are much weaker than latitudinal ones (Figure 6C), most likely reflecting the relative ease with which organisms in Eurasia can move along the east-west axis. The spread of *A. thaliana* and other weedy species may have been further enhanced by the rapid expansion of agriculture along this axis (François et al., 2008). Of particular interest in this respect are unusual populations, such as those in North America, which was colonized only a few centuries ago, and in Central Asia, where reduced genetic differentiation suggests rapid expansion over large geographic distances from a few very small and remote glacial refugia.

#### A High-Quality Community Resource

Questions one can address with natural accessions of *A. thaliana* include how patterns of genetic and epigenetic diversity arose and which forces drive adaptation to the environment. In addition, our knowledge of fundamental molecular processes can be greatly improved through the study of natural change-of-function alleles (Weigel and Nordborg, 2015). Crucial for these purposes is a well characterized, curated, and publicly available collection of accessions. We provide such a collection. Using it as a starting point, increasingly detailed information about (epi)genomes and molecular and non-molecular phenotypes can now be generated. This is a sharp distinction from similar efforts in outcrossing organisms, in which immortalized genotypes are either only available as cell lines, or do not represent adapted genotypes sampled from nature.

The selection of accessions for ecological field studies and laboratory experiments should take into account their full genetic backgrounds. No subset will be optimal for all purposes. A more diverse sample will contain more genetic heterogeneity, which reduces genetic mapping power but captures more variants. The other extreme is represented by the lineage that has recently colonized North America; while it is phenotypically quite uniform, its low diversity provides an opportunity to study the role of de novo variation in adaptation. One should also take into account the natural history of accessions, including local ecology and climate, which may enable informed decisions about phenotypic variation that is likely to reflect adaptation. For example, temperature and precipitation vary greatly across the species’ range and between groups (Figure S6), and one would expect differences in physiological and developmental responses of Spanish and Swedish accessions.

Few, if any, systems offer the benefits of *A. thaliana*: a myriad of sequenced, clonal lineages from a range of ecologically diverse habitats, with patterns of linkage disequilibrium favorable for GWAS, all in an experimentally tractable organism. Another dimension can now be added to traditional functional genomics databases: adaptive variation. The 1001 Genomes collection provides an outstanding opportunity to decipher how genetic variation translates into phenotypic variation and to study the many ways in which plants respond—and have responded—to environmental challenges.

## Experimental Procedures

### Sequencing and Primary Analysis

We initially selected 1,227 worldwide accessions based on genotyping (Platt et al., 2010, Horton et al., 2012) and geographic diversity (Beck et al., 2008, Brennan et al., 2014, Hagmann et al., 2015). They were sequenced by Weigel (MPI), Nordborg (GMI), Ecker (Salk), Mott (Oxford), and Monsanto. Bergelson (University of Chicago) generated the bulk of the seed and tissue used. Paired-end (PE) sequencing employed several generations of the Illumina platform: 1.3+ (80 accessions), 1.5+ (396 accessions), and 1.8+ (751 accessions).

Variants were called with MPI-SHORE (Ossowski et al., 2008) and GMI-GATK (v1.6-5, DePristo et al., 2011) pipelines, validated in our pilot studies (Cao et al., 2011, Long et al., 2013). We generated intersection VCF files with high quality in both pipelines. A series of quality checks resulted in a final set of 1,135 accessions, used for further analyses unless mentioned differently. Variant calls were benchmarked using whole-genome alignments of one long read (Pacific Biosciences) and three short read (Illumina) de novo assemblies against the TAIR10 reference. The average true positive rate (TPR) was 98%, the average false negative rate (FNR) 1.5%, the false discovery rate (FDR) 3%, independent of coverage depth used for the variant calls (Table S5). Pseudogenomes were generated by combining reference and variant calls, including indels.

### Population Genetic Analyses

Please see Supplemental Experimental Procedures for details.

### Data Release

Data and tools are available at http://1001genomes.org. We uploaded raw reads in FASTQ format for 1,135 final accessions to NCBI SRA (SRP056687). We are releasing the following files at http://1001genomes.org/data/GMI-MPI/releases/v3.1: full VCF variant files for each accession, VCF files with quality reference calls, a combined Full Genome VCF file for all genomes, a standard merged group VCF file without invariant positions, a variant annotated SnpEff VCF file, and individual pseudogenome files. Several tools to facilitate the use of this data are available under http://tools.1001genomes.org, including a strain ID web application, a viewer pf ADMIXTURE group membership, and a tool to retrieve specific regions of pseudogenomes in FASTA. Accession metadata, including group membership, are available under http://1001genomes.org/tables/1001genomes-accessions.html. See supplemental data release for additional tools and datasets.

## Consortia

The members of The 1001 Genomes Consortium for this project are Carlos Alonso-Blanco, Jorge Andrade, Claude Becker, Felix Bemm, Joy Bergelson, Karsten M. Borgwardt, Jun Cao, Eunyoung Chae, Todd M. Dezwaan, Wei Ding, Joseph R. Ecker, Moises Exposito-Alonso, Ashley Farlow, Joffrey Fitz, Xiangchao Gan, Dominik G. Grimm, Angela M. Hancock, Stefan R. Henz, Svante Holm, Matthew Horton, Mike Jarsulic, Randall A. Kerstetter, Arthur Korte, Pamela Korte, Christa Lanz, Cheng-Ruei Lee, Dazhe Meng, Todd P. Michael, Richard Mott, Ni Wayan Muliyati, Thomas Nägele, Matthias Nagler, Viktoria Nizhynska, Magnus Nordborg, Polina Yu. Novikova, F. Xavier Picó, Alexander Platzer, Fernando A. Rabanal, Alex Rodriguez, Beth A. Rowan, Patrice A. Salomé, Karl J. Schmid, Robert J. Schmitz, Ümit Seren, Felice Gianluca Sperone, Mitchell Sudkamp, Hannes Svardal, Matt M. Tanzer, Donald Todd, Samuel L. Volchenboum, Congmao Wang, George Wang, Xi Wang, Wolfram Weckwerth, Detlef Weigel, Xuefeng Zhou.

## Author Contributions

J.B., J.R.E., M.No., M.S., and D.W. coordinated the project. C.A.-B., C.B., J.B., J.C., E.C., T.M.D., J.R.E., A.M.H., S.H., M.H., A.K., P.K., N.W.M., M.Na., T.N., M.No., P.N., F.X.P., B.A.R., K.J.S., F.G.S., M.M.T., D.T., W.W., and D.W. selected and generated the samples. C.B., J.B., J.C., X.G., C.L., B.A.R., M.J., R.A.K., T.P.M., R.M., V.N., R.J.S., F.G.S., M.S., S.L.V., and X.Z. generated and handled sequence data. J.A., F.B., A.F., D.G.G., D.M., P.Y.N., A.P., F.A.R., A.R., C.W., and X.W. performed primary sequence analyses and variant annotation. M.E.-A., W.D., A.F., A.M.H., S.R.H., M.H., A.K., C.-R.L., M.No., H.S., and G.W. performed population genetic analyses. J.F., P.K., A.K., A.P., Ü.S., and C.W. curated the online resources and databases. K.M.B., D.G.G., A.K., M.No., and P.A.S. performed GWAS. M.E.-A., A.F., F.B., A.M.H., M.H., A.K., C.-R.L., M.No., A.P., H.S., C.W., G.W., and D.W. wrote the manuscript.

## Accession Numbers

Seeds from direct progeny or siblings of sequenced individuals were deposited with the *Arabidopsis* Biological Resource Center (ABRC), where these were multiplied once. The entire set of accessions is available under accession ID CS78942. The raw sequencing reads for the sequenced individuals have been uploaded in FASTQ format to NCBI SRA (SRP056687).

## Supplemental Information

Document S1. Supplemental Experimental Procedures, Tables S1–S5, and List of The 1001 Genomes Consortium Members and Affiliations

Document S2. Article plus Supplemental Information
