# Supplementary material for: 1,135 Genomes Reveal the Global Pattern of Polymorphism in Arabidopsis thaliana
Source: Cell. 2016 Jul 14;166(2):481–91. doi: 10.1016/j.cell.2016.05.063 (PMC4949382; doi:10.1016/j.cell.2016.05.063)
Supplement: Document S1. Supplemental Experimental Procedures, Tables S1–S5, and List of The 1001 Genomes Consortium Members and Affiliations [file mmc1.pdf]

Cell, Volume *166*

Supplemental Information

1,135 Genomes Reveal the Global Pattern of Polymorphism in *Arabidopsis thaliana*

The 1001 Genomes Consortium

EXTENDED EXPERIMENTAL PROCEDURES

Variant calling

We used the MPI-SHORE and GMI-GATK pipelines, validated in our pilot studies (Cao et al., 2011; Long et al., 2013). Briefly, for the GMI-GATK pipeline, PE reads were mapped to the *Arabidopsis thaliana* TAIR10 reference genome with BWA (v0.5.9-r16) (Li and Durbin, 2009). Format conversions and removal of duplicated reads were done with Samtools (v0.1.6 (r453)) (Li et al., 2009). Local realignment around indels was performed with GATK (v1.6-5) (DePristo et al., 2011) as follows: a first round of indels, called with the UnifiedGenotyper function, was provided to the RealignerTargetCreator function to generate the set of intervals required by the IndelRealigner function. SNPs and indels were called separately, and individually for each accession with the UnifiedGenotyper function, and later merged with the function CombineVariants. Transposons were called from the source data with TE-locate with a resolution of 1000 bp and a minimal read-pair support of 5 (parameters 'minimal Distance to count' = 1000 and 'minimal supporting reads' = 5) (Platzer et al., 2012).

For the MPI-SHORE pipeline (Ossowski et al., 2008), each accession was analyzed separately, using BWA sample (v0.6.2) with option "-n 0.1" to map the reads to the TAIR10 *Arabidopsis thaliana* reference genome sequence. SNPs and short indels were called with SHORE consensus. The matrix with empirically determined penalties for various alignment features from (Cao et al., 2011) was used to calculate the quality score for consensus calls. Features included alignment repetitiveness, absolute and observed-to-expected coverage, allele frequency, base qualities, sequence complexity, GC content, probability of misaligned indels and local coverage uniformity upstream (Cao et al., 2011). Positions with SHORE quality >= 25 and minimum allele frequency of alternative base call >= 0.9 were retained as variant SNP/indel or reference calls. A SHORE2VCF script was developed to convert the SHORE specific quality values into the standard Variant Call Format (VCF) v4.1 (Danecek et al., 2011). In addition to the standard VCF file from SHORE, we also generated a quality reference VCF file with information only from SHORE quality reference calls (Ossowski et al., 2008).

Both the GMI-GATK and the MPI-SHORE pipeline produced standards VCF file for SNPs and short indels for each accessions. For the intersection VCF files, only calls that were in agreement between the two pipelines and that had a quality value >= 25 were accepted. Each intersected VCF file was integrated with the corresponding SHORE quality reference VCF file, which was then used as input for a Full Genome VCF file with the VCF merge tool (Danecek et al., 2011). A standard merged groups VCF file was generated after removing reference calls. SnpEff was used to annotate the standard merged group VCF file (Cingolani et al., 2012), which yielded the variant annotated SnpEff VCF file. All VCF files meet the standard VCF v4.1 (Danecek et al., 2011).

Chloroplast and mitochondrial variants were called using UnifiedGenotyper in GATK with ploidy set to 1, from the same BAM files generated by the GMI-GATK pipeline. Only biallelic SNPs were retained with a quality score >= 100, coverage >= 10x and with >= 75% of reads supporting the alternate allele. Mitochondrial and chloroplastic DNA copy number was estimated as the median coverage normalized by the median coverage of chr1:1-10Mb.

Quality control and validation

Sequence coverage, as determined by read depth at mapped positions, ranged between 2-118X, with 1209 lines having at least 5x and 1058 lines having at least 10x coverage. Read lengths varied from 36 bp to 143 bp, with 1097 lines having reads at least 75 bp long, and 894

lines at least 100 bp. We excluded all non-reference accessions that did not meet the following criteria from the final set: at least 5x coverage; at least 100,000 SNP calls relative to the reference; at least 80% overlap in SNPs between the GMI-GATK and MPI-SHORE pipelines; at least 50% of SNPs called homozygous in both pipelines; contradictions between the two pipelines less than 0.1%; at least 95% concordance with RegMap 250k SNP array data, unless there were reasons to believe that the 250k array data were problematic (Horton et al., 2012). Where more than one lab had sequenced the same accession, the highest quality accession was kept. Finally, accessions with doubtful geographic origin, based on clustering of whole-genome data, were removed. This resulted in a final set of 1135 accessions. Unless mentioned specifically, all analyses were based on this set.

For quality control, we *de novo* assembled three genomes using Illumina reads, from accessions L*er*-1, Ws-2 and Sha. We produced three sequence data sets for each strain: Illumina MiSeq overlapping paired-end reads (400 bp inserts, 250 bp reads, 80x-114x coverage); Illumina HiSeq 2000 mate-pair reads (7 kb inserts, 101 bp reads, 36x-158x coverage); Illumina HiSeq 2000 fosmid-end reads (40 kb inserts, 101 bp reads, 18x-86x coverage). We ran *ALLPATHS-LG* (Gnerre et al., 2011) on a combination of these datasets. We further upgraded the L*er*-1 assembly by filling and reducing scaffold gaps with Ler-1 PacBio reads (PacBio P2C2; 3.4 kb mean/2.2 kb median reads, 240x coverage). We also exploited a more recent Pacific Biosciences L*er*-1 assembly (http://datasets.pacb.com.s3.amazonaws.com/2014/Arabidopsis/reads/list.html). All *de novo* assembled genomes were aligned against the TAIR10 reference using dnadiff (Kurtz et al., 2004). Variants were called directly from whole genome alignments (WGA) using show-snps. Only one-to-one alignments with an identity of at least 90% were taken into account. WGA-based variant calls were compared with variant calls from our final 1001G dataset, bsed on combining the results from the MPI-SHORE and GMI-GATK pipelines. Indels were left-aligned and normalized prior to the comparison using the norm function from bcftools. True Positives (TP) are positions where 1001G and WGA variants are concordant, False Positives (FP) where 1001G variants were not supported by WGA (which may either show a reference call or a different variant), False Negatives (FN) where a 1001G reference call is not supported by WGA (which has a variant). The False Discovery Rate (FDR) is defined as FP/(TP+FP), the False Negative Rate is defined as FN/(FN+TP) and the True Positive Rate is defined as TP/(TP+FN). Comparing the combined variant calls with the WGA-based variant calls resulted in an average TPR of 98%, an average FNR of 1.5% and an average FDR of 3%. TPR and FDR were further monitored over a range of different coverage depths (5-45x coverage) by randomly subsetting a L*er*-1 dataset to call variants with the two pipelines against TAIR10 as described above. FDR (3±0.4%), FNR (1.5±0.1%) and TPR (98±0.1%) remained independent of the coverage depth (5-45x coverage) (Table S5).

Pseudogenomes and variant annotation

Pseudogenomes were generated by combining reference and variant calls including indels, with uncalled sites represented as Ns, plus an index for finding Col-0 annotated regions.

Variants were annotated with SnpEff (release 4.1L) and the SnpEff *A. thaliana* database (release 2015-01-08) (Cingolani et al., 2012). Derived alleles were extracted from a three way alignment of *A. thaliana* (TAIR10), *A. lyrata* and *C. orientalis* (unpublished PacBio assemblies) calculated with progressive Cactus (Paten et al., 2011a, 2011b). Sub-alignments with more than one sequence from one of the species, or one of the species missing were discarded. The remaining alignments were screened for identical sites. 35 Mb of the *A. thaliana* genome was

marked as derived in this way. Allele density spectrums were smoothed and plotted with the sm package in GNU R (Lenth, 2009).

Genome-wide association studies

Seeds for all 1135 accessions were surface-sterilized with chlorine gas. Seeds were distributed in pots with four replicates in a randomized block design, each replicate corresponding to one block. Plants were grown in growth chambers with the following settings: after 6 days of stratification in the dark at 4ºC, constant temperature of 10°C or 16°C with 16 hours light / 8 hours darkness, 65% humidity. All trays within a block were moved to a new shelf and rotated 180º every other day to minimize position effects. Flowering time was scored as days until first open flower. Genome-wide association mapping was done on the means of the four replicates for both Phenotypes (10°C and 16°C) independently, using an approximation of the mixed model that has been described previously (Kang et al., 2010, pipeline available at: https://github.com/arthurkorte/GWAS).

We compared the 1001G SNPS to the 250k SNP-array data from Horton et al. (2012). Out of the 214,051 SNPs called with the 250k array, 207,096 are called as SNPs in the 1001G data, with 192,498 (93.0%) being biallelic, and 14,598 (7.0%) multi-allelic. For 530 of the biallelic SNPs, the inferred state between the two datasets differs. These are distributed across all chromosomes according to local SNP density (Supplemental Figure S7) and are unlikely to negatively affect GWAS (Supplemental Fig S8). In general, given the much higher estimated error rates in the 250k SNP-array data (Atwell et al. 2010; Horton et al., 2012), disagreement with the 1001G data are likely to result from errors in the 250k SNP-array data (Supplemental Figure S9).

Population genetic analyses

We used Beagle v3 (Browning and Browning, 2009) to impute missing SNPs based on linkage disequilibrium with default parameters, followed by GERMLINE v1.5.1 (Gusev et al., 2009) for error-tolerant and computationally efficient identification of Identity-by-Descent (IBD) regions on these imputed SNPs. Pairwise IBD segments were detected as long continuous stretches with a minimum length of 10 kb, merged from slices containing 100 identical SNPs and allowing for maximally two mismatches.

MSMC input was created parsing a VCF file including all sites (variant and non-variant). Filtered sites or sites were any of the focal individuals had missing genotypes were excluded from the count of "called sites". MSMC was run in the two haplotype mode with the option --fixedRecombination, were haplotypes of different inbred individuals were used together. Scaled times were converted to years assuming a generation time of one year and a mutation rate of 7*10^(-9) (Ossowski et al., 2010). Coalescent rate was calculated as 1/(relative effective population size).

To infer the relationship between relicts and non-relicts for all individual genes, we estimated the genealogy between two non-relict and two relict accessions. This analysis was done separately for each pairs of Iberian relicts while fixing Col-0 and L*er*-0 as the two non-relicts in the 4-taxon test. From the start of each gene, we searched for non-recombining region by performing four-gamete tests for all consecutive SNP pairs until a recombination event among the four accessions was detected. Gene genealogy was estimated from the phylogenetically informative sites (doubletons) within the non-recombining region, and genes with less than two informative sites were excluded.

Two additional four-taxon gene tree analyses were also conducted. One with *A. lyrata*, *A. thaliana* Col-0 and two relicts, and the other with *A. lyrata*, *A. thaliana* Col-0, one relict, and one non-relict. We used CONSEL (Shimodaira and Hasegawa, 2001) and RAxML (Stamatakis, 2014) to generate Maximum Likelihood gene trees, and determined their significance with the AU test (significance threshold 0.05) (Shimodaira, 2002). The deviation from null distribution was assessed for each chromosome via one-tailed Student's t-tests.

SPA geographic projections and SNP gradient analyses were performed using SPA v1.13 (Yang et al. 2012), with default parameters. One-tailed *p*-values were calculated by first transforming SPA scores to *z*-scores.

Climate data (both historical and recent) were obtained from WorldClim (www.worldclim.org). Recent data were extracted from the Current Conditions Bioclim rasters (Hijmans et al. 2005, http://www.worldclim.org/current). Historical data were extracted from the CMIP5 Multi-Model Ensemble dataset (MPI-ESM-P, http://www.worldclim.org/paleo-climate). Data was sourced from the 30 arc-second rasters, with 2.5 arc-minute rasters as a fallback if collection locations fell between raster cells.

We used a mixed model approach to identify variants associated with latitude and six representative Bioclim variables, while controlling for the potentially confounding effects of population structure. The Bioclim variables included in the analysis were annual mean temperature, annual precipitation, mean temperature during warmest quarter, mean temperature during the coldest quarter, precipitation in the wettest quarter and precipitation during the driest quarter. We used GEMMA (Zhou and Stephens, 2012) to infer the correlation between each variant with frequency >5% in the total sample and each of the seven variables. First, we estimated a relatedness matrix for each chromosome using the '-gk 1' option in GEMMA. Then, we assessed evidence for correlation in a linear mixed model framework using Rao's score test. We identified the set of variants with a false discovery rate <5% for each variable (R package: p.adjust, method="fdr") and used a comparison to the closest outgroup relative, *A. lyrata*, to determine the ancestral state.

To identify candidate selective sweeps, we used OmegaPlus (Kim and Nielsen, 2004; Alachiotis et al., 2012). The grid size was chosen to ensure the statistic was evaluated, on average, every 500 bp while the minimum and maximum regions considered were 10-kb and 100-kb, respectively. For the F_{ST} scan, Weir and Cockerham's (Weir and Cockerham, 1984) was calculated at each SNP, to identify genomic regions that have diverged among these groups. To identify GO terms (TAIR) overrepresented in the top 1% results from the F_{ST} scan, which were summarized in 10-kb windows, we omitted gene-models with low confidence (evidence-code: 'inferred from electronic annotation') and any biological category represented by only one gene-model. We used Storey's approach (Storey and Tibshirani, 2003) to correct for multiple testing.

Data release

Although some of the original data have been released in conjunction with prior publications (Cao et al., 2011; Gan et al., 2011; Schneeberger et al., 2011; Schmitz et al., 2013; Long et al., 2013; Hagmann et al., 2015), we uploaded raw reads in fastq format for all 1135 final accessions to NCBI SRA with id SRP056687. We are releasing the full VCF variant files from the intersection of the GMI (GATK) and MPI (SHORE) pipelines for each accession on the http://1001genomes.org project website under

http://1001genomes.org/data/GMI-MPI/releases/v3.1/intersection_snp_short_indel_vcf/.

We also produced VCF files that include information on quality reference calls:
http://1001genomes.org/data/GMI-MPI/releases/v3.1/intersection_snp_short_indel_vcf_with_quality_reference/.

The combined Full Genome VCF file that includes information for all genomes (132 GB):
http://1001genomes.org/data/GMI-MPI/releases/v3.1/1001genomes_snp-short-indel_with_tair10_only_ACGTN.vcf.gz.

The standard merged group VCF file without information on invariant positions (~18 GB):
http://1001genomes.org/data/GMI-MPI/releases/v3.1/1001genomes_snp-short-indel_only_ACGTN.vcf.gz.

The variant annotated SnpEff VCF file (~17 GB):
http://1001genomes.org/data/GMI-MPI/releases/v3.1/1001genomes_snpeff_v3.1/1001genomes_snp-short-indel_only_ACGTN_v3.1.vcf.snpeff.gz.

Individual pseudogenome files in gzipped FASTA format:
http://1001genomes.org/data/GMI-MPI/releases/v3.1/pseudogenomes/.

The imputed SNP matrix (317 MB):
http://1001genomes.org/data/GMI-MPI/releases/v3.1/SNP_matrix_imputed_hdf5/1001_SNP_MATRIX.tar.gz.

Accession metadata, including group membership:
http://1001genomes.org/tables/1001genomes-accessions.html

The phenotypes for flowering time scored at 10°C and 16°C:
http://1001genomes.org/tables/1001genomes-FT10-FT16_and_1001genomes-accessions.html

We created several tools to facilitate using this dataset under http://tools.1001genomes.org.
Our strain ID web application:
http://tools.1001genomes.org/strain_id.

A tool to download specific regions of psuedogenomes:
http://tools.1001genomes.org/pseudogenomes.

Online visualization tool to view ADMIXTURE group membership and genetic composition:
http://1001genomes.github.io/admixture-map.

SUPPLEMENTAL TABLES

Table S1. Gene tree analyses, Related to Figure 4.

Mean and standard deviation of genes with resolved four-sample topology supporting the expected or unexpected relationship among Col-0, Ler-0, and two Iberian relicts.

	**Gene count**	**Proportion**
Concordant	3867.23 (+- 968.83)	0.71 (+- 0.11)
Discordant	1327.98 (+- 383.91)	0.26 (+- 0.10)

Table S2. SNPs from the climate correlation analysis with FDR<0.05. Bold denotes nonsynonymous variants, Related to Figure 5.

Variable	Chr	Position	Frequency	p-value	GeneID	Gene Name
Annual precipitation	3	8201102	0.057	3.14E-07	AT3G23060	CYP705A33
	**4**	**13187833**	0.108	3.48E-08	AT4G25970	PSD3
	4	13191070	0.108	3.72E-08	AT4G25980	
	5	17883475	0.069	4.26E-07	AT5G44390	
	5	17883492	0.069	3.50E-07	AT5G44390	
	5	17883501	0.067	4.22E-07	AT5G44390	
	5	17883508	0.068	4.79E-07	AT5G44390	
	5	21818955	0.066	5.09E-07	Intergenic	
	5	21818969	0.068	4.11E-07	Intergenic	
Precipitation wettest quarter	1	1697341	0.081	9.48E-07	Intergenic	
	1	16373400	0.1	8.67E-07	AT1G43387	TE gene
	3	334271	0.09	5.46E-07	Intergenic	
	3	7341449	0.128	1.23E-07	AT3G20940	CYP705A30
	**3**	**7341468**	0.123	6.43E-07	AT3G20940	CYP705A30
	**3**	**7345743**	0.101	6.04E-08	AT3G20960	CYP705A33
	3	8296396	0.209	3.44E-07	AT3G23240	ERF1
	**4**	**9814059**	0.083	1.58E-06	AT4G17610	tRNA/rRNA methyltransferase
	**4**	**13187833**	0.108	8.75E-07	AT4G25970	PSD3
	4	13191070	0.108	5.22E-07	AT4G25980	
	5	9063610	0.048	5.55E-10	AT5G44390	FAD-binding
	5	9072657	0.07	2.95E-07	Intergenic	
	5	17883475	0.069	2.14E-07	AT5G44390	
	5	17883476	0.069	1.10E-06	AT5G44390	
	5	17883492	0.069	3.38E-07	AT5G44390	
	5	17883501	0.067	3.61E-07	AT5G44390	

	5	17883508	0.068	1.28E-07	AT5G44390
	5	21818969	0.068	1.33E-06	Intergenic

Table S3. IDs of Iberian relicts and their closest matching (unique) non-relict, Related to Figure 5.

Iberian relict	Iberian non-relict	Haversine distance (km)
9832	9862	10.15
9837	9873	13.15
9947	9855	18.92
9533	9531	19.58
9871	9841	22.33
9905	9843	26.68
9542	9822	30.13
9869	9522	26.70
9600	9943	28.90
9543	9900	87.33
9598	9578	31.93
9555	9556	36.23
9545	9544	38.67
9550	9590	36.89
9887	9534	40.23
9549	9903	49.35
9554	6961	66.95
9944	9537	140.40
9574	9514	170.62
9583	9541	221.71
9879	9518	261.95

Table S4. Top five significant Gene Ontology (GO) terms enriched in relicts compared to geographically close non-relicts. Ties among *q* - values are ranked according to results from Fisher's Exact test, Related to Figure 5.

Rank	Biological Process	Enrichment	FDR q
1	Flower development	6.3	0.00059
2	Positive regulation of abscisic acid mediated signaling pathway	15.4	0.00059
3	Embryo sac development	7.7	0.00059
4	Embryo development	6.2	0.00059
5	Positive regulation of flower development	9.5	0.00059

Table S5. Error rate dependencies, Related to Experimental Procedures.

Error rates as a function of sequencing depth and genomic context based on read data from a single accession (L*er*).

Coverage/ Annotation	TP	FP	FN	TPR	FNR	FDR
5	185,402	4,424	3,042	98.39%	1.61%	2.33%
7	314,276	7,504	4,246	98.67%	1.33%	2.33%
9	373,409	9,233	4,982	98.68%	1.32%	2.41%
12	404,520	10,668	5,549	98.65%	1.35%	2.57%
14	423,364	11,711	5,995	98.60%	1.40%	2.69%
17	434,153	12,463	6,286	98.57%	1.43%	2.79%
20	443,682	13,168	6,504	98.56%	1.44%	2.88%
22	450,936	13,805	6,732	98.53%	1.47%	2.97%
24	456,516	14,303	6,907	98.51%	1.49%	3.04%
27	461,342	14,815	7,036	98.50%	1.50%	3.11%
29	465,272	15,269	7,199	98.48%	1.52%	3.18%
32	468,692	15,661	7,285	98.47%	1.53%	3.23%
34	471,446	16,085	7,397	98.46%	1.54%	3.30%
37	474,048	16,441	7,399	98.46%	1.54%	3.35%
39	476,257	16,710	7,494	98.45%	1.55%	3.39%
41	478,062	16,904	7,618	98.43%	1.57%	3.42%
45	479,863	17,140	7,702	98.42%	1.58%	3.45%
5’ UTR	22,823	208	245	98.94%	1.06%	0.90%
3’ UTR	28,400	381	281	99.02%	0.98%	1.32%
Exon	143,361	2,201	1,202	98.52%	0.83%	1.51%
Intron	81,572	1,176	1,126	98.64%	1.36%	1.42%
Intergenic	264,123	9,410	3,485	96.62%	2.05%	4.99%
Repetitive	99,705	9,410	3,485	96.62%	3.38%	8.62%
Non-repetitive	380,158	7,730	4,217	98.90%	1.10%	1.99%

SUPPLEMENTAL REFERENCES

Alachiotis, N., Stamatakis, A., and Pavlidis, P. (2012). OmegaPlus: a scalable tool for rapid detection of selective sweeps in whole-genome datasets. Bioinformatics *28*, 2274-2275.

Browning, B.L., and Browning, S.R. (2009). A unified approach to genotype imputation and haplotype-phase inference for large data sets of trios and unrelated individuals. Am. J. Hum. Genet. *84*, 210-223.

Cao, J., Schneeberger, K., Ossowski, S., Günther, T., Bender, S., Fitz, J., Koenig, D., Lanz, C., Stegle, O., Lippert, C.*, et al.* (2011). Whole-genome sequencing of multiple *Arabidopsis thaliana* populations. Nat Genet *43*, 956-963.

Cingolani, P., Platts, A., Wang le, L., Coon, M., Nguyen, T., Wang, L., Land, S.J., Lu, X., and Ruden, D.M. (2012). A program for annotating and predicting the effects of single nucleotide polymorphisms, SnpEff: SNPs in the genome of Drosophila melanogaster strain w^{1118}*; iso-2; iso-3*. Fly *6*, 80-92.

Danecek, P., Auton, A., Abecasis, G., Albers, C.A., Banks, E., DePristo, M.A., Handsaker, R.E., Lunter, G., Marth, G.T., Sherry, S.T., et al. (2011). The variant call format and VCFtools. Bioinformatics *27*, 2156-2158.

DePristo, M.A., Banks, E., Poplin, R., Garimella, K.V., Maguire, J.R., Hartl, C., Philippakis, A.A., del Angel, G., Rivas, M.A., Hanna, M.*, et al.* (2011). A framework for variation discovery and genotyping using next-generation DNA sequencing data. Nat Genet *43*, 491-498.

Gan, X., Stegle, O., Behr, J., Steffen, J.G., Drewe, P., Hildebrand, K.L., Lyngsoe, R., Schultheiss, S.J., Osborne, E.J., Sreedharan, V.T.*, et al.* (2011). Multiple reference genomes and transcriptomes for *Arabidopsis thaliana*. Nature *477*, 419-423.

Gnerre, S., Maccallum, I., Przybylski, D., Ribeiro, F.J., Burton, J.N., Walker, B.J., Sharpe, T., Hall, G., Shea, T.P., Sykes, S., et al. (2011). High-quality draft assemblies of mammalian genomes from massively parallel sequence data. Proc. Natl. Acad. Sci. USA *108*, 1513-1518.

Gusev, A., Lowe, J.K., Stoffel, M., Daly, M.J., Altshuler, D., Breslow, J.L., Friedman, J.M., and Pe'er, I. (2009). Whole population, genome-wide mapping of hidden relatedness. Genome Res *19*, 318-326.

Hagmann, J., Becker, C., Müller, J., Stegle, O., Meyer, R.C., Wang, G., Schneeberger, K., Fitz, J., Altmann, T., Bergelson, J.*, et al.* (2015). Century-scale methylome stability in a recently diverged *Arabidopsis thaliana* lineage. PLoS Genet. *11*, e1004920.

Hijmans, R.J., Cameron, S.E., Parra, J.L., Jones, P.G., and Jarvis, A. (2005). Very high resolution interpolated climate surfaces for global land areas. Int. J. Climat. *25*, 1965-1978.

Horton, M., Hancock, A.M., Huang, Y.S., Toomajian, C., Atwell, S., Auton, A., Muliyati, W., Platt, A., Sperone, F.G., Vilhkálmsson, B.J.*, et al.* (2012). Genome-wide pattern of genetic variation in worldwide *Arabidopsis thaliana* accessions from the *RegMap* panel. Nat Genet *44*, 212-216.

Kang, H.M., Sul, J.H., Service, S.K., Zaitlen, N.A., Kong, S.Y., Freimer, N.B., Sabatti, C., and Eskin, E. (2010). Variance component model to account for sample structure in genome-wide association studies. Nat. Genet. *42*, 348-354.

Kim, Y., and Nielsen, R. (2004). Linkage disequilibrium as a signature of selective sweeps. Genetics *167*, 1513-1524.

Kurtz, S., Phillippy, A., Delcher, A.L., Smoot, M., Shumway, M., Antonescu, C., and Salzberg, S.L. (2004). Versatile and open software for comparing large genomes. Genome Biol. *5*, R12.

Lenth, R.V. (2009). Response-surface methods in R, using rsm. J. Stat. Software *32*.

Li, H., and Durbin, R. (2009). Fast and accurate short read alignment with Burrows-Wheeler transform. Bioinformatics *25*, 1754-1760.

Li, H., Handsaker, B., Wysoker, A., Fennell, T., Ruan, J., Homer, N., Marth, G., Abecasis, G., and Durbin, R. (2009). The Sequence Alignment/Map format and SAMtools. Bioinformatics *25*, 2078-2079.

Long, Q., Rabanal, F.A., Meng, D., Huber, C.D., Farlow, A., Platzer, A., Zhang, Q., Vilhjálmsson, B.J., Korte, A., Nizhynska, V.*, et al.* (2013). Massive genomic variation and strong selection in *Arabidopsis thaliana* lines from Sweden. Nat. Genet. *45*, 884-890.

Ossowski, S., Schneeberger, K., Clark, R.M., Lanz, C., Warthmann, N., and Weigel, D. (2008). Sequencing of natural strains of *Arabidopsis thaliana* with short reads. Genome Res *18*, 2024-2033.

Ossowski, S., Schneeberger, K., Lucas-Lledo, J.I., Warthmann, N., Clark, R.M., Shaw, R.G., Weigel, D., and Lynch, M. (2010). The rate and molecular spectrum of spontaneous mutations in *Arabidopsis thaliana*. Science *327*, 92-94.

Paten, B., Diekhans, M., Earl, D., John, J.S., Ma, J., Suh, B., and Haussler, D. (2011a). Cactus graphs for genome comparisons. J. Comput. Biol. *18*, 469-481.

Paten, B., Earl, D., Nguyen, N., Diekhans, M., Zerbino, D., and Haussler, D. (2011b). Cactus: Algorithms for genome multiple sequence alignment. Genome Res. *21*, 1512-1528.

Platzer, A., Nizhynska, V., and Long, Q. (2012). TE-Locate: A tool to locate and group transposable element occurrences using paired-end next-generation sequencing data. Biology *1*, 395-410.

Qi, J., Liu, X., Shen, D., Miao, H., Xie, B., Li, X., Zeng, P., Wang, S., Shang, Y., Gu, X., et al. (2013). A genomic variation map provides insights into the genetic basis of cucumber domestication and diversity. Nat. Genet. *45*, 1510-1515.

Schmitz, R.J., He, Y., Valdes-Lopez, O., Khan, S.M., Joshi, T., Urich, M.A., Nery, J.R., Diers, B., Xu, D., Stacey, G.*, et al.* (2013). Epigenome-wide inheritance of cytosine methylation variants in a recombinant inbred population. Genome Res. *23*, 1663-1674.

Schneeberger, K., Ossowski, S., Ott, F., Klein, J.D., Wang, X., Lanz, C., Smith, L.M., Cao, J., Fitz, J., Warthmann, N.*, et al.* (2011). Reference-guided assembly of four diverse *Arabidopsis thaliana* genomes. Proc. Natl. Acad. Sci. USA *108*, 10249-10254.

Shimodaira, H. (2002). An approximately unbiased test of phylogenetic tree selection. Syst. Biol. *51*, 492-508.

Shimodaira, H., and Hasegawa, M. (2001). CONSEL: for assessing the confidence of phylogenetic tree selection. Bioinformatics *17*, 1246-1247.

Stamatakis, A. (2014). RAxML version 8: a tool for phylogenetic analysis and post-analysis of large phylogenies. Bioinformatics *30*, 1312-1313.

Storey, J.D., and Tibshirani, R. (2003). Statistical significance for genomewide studies. Proc Natl Acad Sci USA *100*, 9440-9445.

Weir, B.S., and Cockerham, C.C. (1984). Estimating F-statistics for the analysis of population structure. Evolution *38*, 1358-1370.

Yang, W.Y., Novembre, J., Eskin, E., and Halperin, E. (2012). A model-based approach for analysis of spatial structure in genetic data. Nat Genet *44*, 725-731.

Zhou, X., and Stephens, M. (2012). Genome-wide efficient mixed-model analysis for association studies. Nat. Genet. *44*, 821-824.

AUTHOR AFFILIATIONS

Carlos Alonso-Blanco[1], Jorge Andrade[2], Claude Becker[3], Felix Bemm[3], Joy Bergelson[2], Karsten M. Borgwardt[4], Eunyoung Chae[3], Todd Dezwaan[5], Wei Ding[3], Joseph R. Ecker[6], Moises Exposito-Alonso[3], Ashley Farlow[7,8], Joffrey Fitz[3,9], Xiangchao Gan[10], Dominik G. Grimm[3,4], Angela Hancock[2,11,12], Stefan R. Henz[3], Svante Holm[13], Matthew Horton[2,7], Mike Jarsulic[2], Randall A. Kerstetter[14], Arthur Korte[7], Pamela Korte[7], Christa Lanz[3], Cheng-Ruei Lee[7], Dazhe Meng[7], Todd P. Michael[5], Richard Mott[10], Ni Wayan Muliyati[2], Thomas Nägele[12,15], Matthias Nagler[12], Viktoria Nizhynska[7], Magnus Nordborg[7], Polina Yu. Novikova[7], F. Xavier Picó[16], Alexander Platzer[7], Fernando A. Rabanal[7], Alex Rodriguez[2], Beth A. Rowan[3], Patrice A. Salomé[3], Karl Schmid[17], Robert J. Schmitz[6], Ümit Seren[7], Felice Gianluca Sperone[2], Mitchell Sudkamp[14], Hannes Svardal[7], Matt M. Tanzer[5], Donald Todd[5], Samuel L. Volchenboum[2], Congmao Wang[3,18], George Wang[3], Xi Wang[3], Wolfram Weckwerth[12,15], Detlef Weigel[3], Xuefeng Zhou[5]

[1]Centro Nacional de Biotecnología, Consejo Superior de Investigaciones Científicas, Madrid-28049, Spain; [2]Dept. of Ecology and Evolution, University of Chicago, Chicago, IL 60637, USA; [3]Dept. of Molecular Biology, Max Planck Institute for Developmental Biology, 72076 Tübingen, Germany; [4]Dept. of Biosystems Science and Engineering, ETH Zurich, 4058 Basel, Switzerland; [5]Monsanto, Research Triangle Park, NC 27709, USA; [6]Salk Institute for Biological Studies, La Jolla, CA 92037, USA; [7]Gregor Mendel Institute (GMI), Austrian Academy of Sciences, Vienna Biocenter (VBC), 1030 Vienna, Austria; [8]Centre for Systems Genomics, The University of Melbourne, Australia; [9]Tropic IT Ltd., Central, Hong Kong; [10]Wellcome Trust Centre for Human Genetics, University of Oxford, Oxford OX3 7BN, UK; [11]Max F. Perutz Laboratories, 1030 Vienna, Austria; [12]Dept. of Ecogenomics and Systems Biology, University of Vienna, 1090 Vienna, Austria; [13]Department of Natural Sciences, Mid-Sweden University, 851 70 Sundsvall, Sweden; [14]Monsanto, Chesterfield, MO 63017, USA; [15]Vienna Metabolomics Center, University of Vienna, 1090 Vienna, Austria; [16]Estación Biológica de Doñana, Consejo Superior de Investigaciones Científicas, Sevilla-41092, Spain; [17]Institute of Plant Breeding, Seed Science and Population Genetics, University of Hohenheim, 70593 Stuttgart, Germany; [18]Institute of Digital Agriculture, Zhejiang Academy of Agricultural Sciences, Hangzhou, Zhejiang, 310021, PR China

Current addresses:

Center for Research Informatics, The University of Chicago, Chicago, Illinois, USA (JA); Max Planck Institute for Plant Breeding Research, 50829 Cologne, Germany (XG); intomics, 2800 Lyngby, Denmark (SRH); Center for Computational and Theoretical Biology, University Würzburg, 97074 Würzburg, Germany (AK, PK); Ibis Biosciences, Carlsbad, CA 92008, USA (TM); Genetics Institute, University College London, London WC1E 6BT, UK (RM); Dept. of Chemistry and Biochemistry, UCLA, Los Angeles, CA 90095, USA (PAS); Dept. of Genetics, University of Georgia, Athens, GA 30602, USA (RJS); Wellcome Trust Sanger Institute, Hinxton, Cambridgeshire CB10 1SA, UK (HS); Monsanto, Chesterfield, MO 63017, USA (DT); Bayer Cropscience, 9052 Gent, Belgium (XW)
